# piRNA-Mediated Maintenance of Genome Stability in Gametogenesis and Cancer

**DOI:** 10.3390/genes16070722

**Published:** 2025-06-20

**Authors:** Martyna Zawalska, Maciej Tarnowski

**Affiliations:** 1 Department of Physiology in Health Sciences, Faculty of Health Sciences, Pomeranian Medical University in Szczecin, Żołnierska 48, 70-210 Szczecin, Poland; 2 Institute of Physical Culture Sciences, University of Szczecin, 70-453 Szczecin, Poland

**Keywords:** cancer, epigenetic regulation, gametogenesis, genome stability, oogenesis PIWI-interacting RNA, transposable elements

## Abstract

Epigenetics and genome science have become central to current molecular biology research. Among the key mechanisms ensuring genomic integrity is the silencing of transposable elements in germline cells, a process essential for fertility in both sexes. A pivotal component of this silencing machinery involves PIWI-interacting RNAs (piRNAs), a distinct class of small non-coding RNAs that regulate gene expression and suppress transposable elements at both the transcriptional and post-transcriptional levels. piRNAs function in concert with PIWI proteins, whose expression is critical for proper oogenesis, spermatogenesis, and early zygote development. Disruptions in piRNA or PIWI protein pathways not only impair germline function but also contribute to genome instability, unchecked cell proliferation, and aberrant epigenetic modifications, hallmarks of tumorigenesis. Emerging evidence links the dysregulation of the piRNA/PIWI axis to the development and progression of various cancers, including lung and colorectal carcinomas. This review highlights the fundamental roles of piRNAs and PIWI proteins in reproductive biology and their increasingly recognized relevance in cancer biology.

## 1. Introduction

PIWI-interacting RNA (piRNA), a class of small non-coding RNAs [1,2,3,4], plays a crucial role in genome stability by suppressing transposable elements (TEs) and regulating gene expression. piRNA belongs to a class of small RNAs that includes small interfering RNA (siRNA), microRNA (miRNA), and piRNA itself [5,6,7,8]. These small RNAs play essential roles in gene regulatory pathways, helping to maintain genomic integrity. While all small RNAs contribute to gene regulatory pathways and genomic integrity, piRNAs are distinguished by their capacity to guide epigenetic modifications, including DNA methylation and histone remodeling, primarily through their interaction with PIWI proteins [5,6,8]. piRNAs are predominantly expressed in germline cells, where they interact with PIWI proteins to form RNA–protein complexes essential for transposon silencing, DNA methylation, and histone modifications. Recent studies have also revealed piRNA expression in somatic tissues, where they appear to participate in processes such as stem cell maintenance, cellular differentiation, and stress responses. Notably, dysregulation of the piRNA/PIWI pathway exerts particularly profound effects compared to other small RNA classes, due to its upstream regulatory role in chromatin organization and genome stability [8,9].

Interestingly, recent studies have also linked piRNAs and PIWI proteins to cancer development. Dysregulation of the piRNA pathway has been associated with multiple types of cancer, including ovarian, breast [10], and gastrointestinal cancers [11]. The aberrant expression of piRNAs and PIWI proteins can lead to genome instability, uncontrolled cell proliferation, and altered epigenetic landscapes, all of which contribute to tumor progression [8,12,13,14]. Some piRNAs have been identified as tumor suppressors, preventing the activation of oncogenes, while others may function as oncogenic factors, promoting metastasis and resistance to therapy [10,11,12,13,14]. These findings suggest that piRNAs could serve as potential biomarkers for cancer diagnosis and prognosis, as well as novel therapeutic targets for precision medicine [14]. This narrative review aims to investigate the critical roles of PIWI-interacting RNAs (piRNAs) and PIWI proteins in regulating germline cell development and cancer progression and epigenetic pathways. This study provides a detailed description of piRNA molecules, piRNA biogenesis [15,16,17], and PIWI proteins which interact with mRNA. Moreover, we discuss alterations in piRNA/PIWI expression levels and the regulation of piRNA/PIWI pathways, which are vital in both male and female fertility [18,19,20] and in tumorigenesis. This review delves into the biological and pathological significance of the piRNA/PIWI pathway, specifically dissecting its multifaceted roles in both germline development and tumorigenesis. The intricate processes of piRNA biogenesis, the critical molecular interactions between piRNAs and PIWI proteins, and the profound consequences of their dysregulation are examined. The aim of this exploration is to illuminate the dualistic nature of piRNAs in reproduction and cancer, providing comprehensive insight into how a single, fundamental regulatory system can crucially bridge these two seemingly disparate biological contexts.

## 2. Transposable Elements

Transposable elements (TEs), also referred to as “jumping genes” [21,22], are mobile DNA sequences capable of moving within the genome, replicating autonomously, and integrating into new genomic locations [23,24,25]. This mobility poses a potential threat to the stability of an organism’s genetic material. TEs are broadly classified into two major categories based on their transposition mechanisms. Class I elements, or retrotransposons, utilize reverse transcriptase to create a new DNA copy that is then inserted, thereby expanding their population within the genome. Class II elements, or DNA transposons, operate more directly and generally utilize a “cut-and-paste” mechanism, whereby the element is excised from one genomic location and inserted into another—a process catalyzed by the transposase enzyme [23,24]. TEs are present in all studied genomes, and their tight regulation is essential for preserving genomic stability, particularly in germ cells [26,27,28,29]. However, the host defense mechanisms are not capable of recognizing or suppressing every mutation or transposition event. Consequently, some mutations can evade surveillance, potentially becoming heritable and accumulating over generations. In response, the defense systems can adapt to these changes, allowing TEs to remain under control and restore genomic stability. In germline cells, one of the primary regulatory mechanisms controlling TEs is mediated by PIWI-interacting RNAs (piRNAs) [26,27,28,29].

## 3. Origin and Structure of piRNA and PIWI Proteins Family

**Figure 1:**
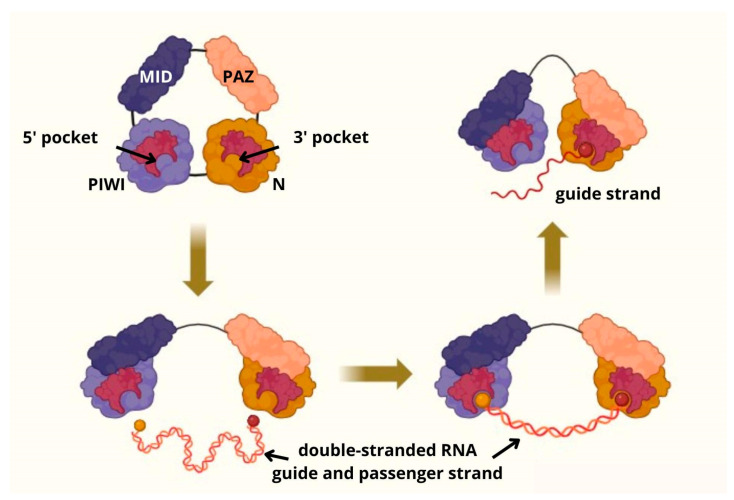
The Argonaute protein family (AGO) contains four domains: N-terminal (N), PIWI-AGO-Zwille (PAZ), Middle (MID), and PIWI domains—and two linkers (L1 and L2). The L1 linker is connected with the N and PAZ domains; in turn, the L2 linker combines the N and PAZ domains with the MID and PIWI domains. The N and PIWI domains feature 5′ and 3′ pockets that bind double-stranded RNA (dsRNA). Subsequently, the passenger strand of dsRNA detaches from the 3′ domain, while the guide strand remains bound to AGO. The PIWI domain is responsible for silencing transposable elements [34,35,36,37,38].

piRNAs are single-stranded RNA molecules of 24–31 nucleotides in length, making them the longest known small RNAs within the class that also includes microRNAs (miRNAs) and small interfering RNAs (siRNAs) [5,8,15,17,25,27,28]. Their primary function is to preserve genomic integrity, primarily by silencing TEs at both the transcriptional and post-transcriptional levels [29,30,31,32]. Post-Transcriptional Gene Silencing (PTGS) is mediated via the Ping-Pong Amplification Cycle, a self-reinforcing loop that degrades transposon transcripts. Transcriptional Gene Silencing (TGS) happens through epigenetic modifications [29,32,33]. piRNAs exert their regulatory effects by forming ribonucleoprotein complexes with PIWI proteins, a subfamily of the Argonaute (AGO) protein family [29,32,33] Figure 1. The AGO family consists of a group of closely related, evolutionary conserved proteins that interact with small non-coding RNAs to form RNA-induced silencing complexes (RISCs). These complexes are guided by small RNAs to complementary target RNAs, resulting in gene silencing through mechanisms such as mRNA cleavage, transcript degradation, and translational repression [32,33,34]. AGO proteins, including the PIWI subfamily, are structurally characterized by four conserved domains: N-terminal (N), PIWI-AGO-Zwille (PAZ), Middle (MID), and PIWI domains—and two linkers (L1 and L2). The L1 linker bridges the N and PAZ domains, while the L2 linker connects the N and PAZ domains with the MID and PIWI domains. The N-terminal domain, located at the protein’s amino terminus, undergoes conformational changes that facilitate the unwinding of small RNA duplexes. The MID and PAZ domains recognize and bind the 5′ end and the 3′ end of the RNA duplex strand. The PIWI domain is responsible for TE silencing [34,35,36,37]. The endonucleolytic “slicer” activity of PIWI proteins is mediated by a conserved DD [E/D] catalytic triad within the PIWI domain, which requires divalent metal ions and specific Asp/Glu residues for proper function. This catalytic center enables precise cleavage of RNA targets bound by the piRNA guide. The MID domain precisely anchors the 5′-phosphate of the guide RNA, while the PAZ domain secures its 3′ end. This coordinated structural arrangement precisely guides the piRNA for optimal target recognition and subsequent downstream effects, including targeted transcript degradation and efficient heterochromatin recruitment. Failures in trimming result in unstable, untrimmed piRNAs that cannot load correctly onto PIWI proteins, leading to transposon derepression and germ cell failure with consequent male infertility. Furthermore, improper 2′-O-methylation at the piRNA 3′ end compromises stability and impairs silencing complex assembly, further weakening the genome defense system [35,36,37,38].

### The piRNA Biogenesis Pathway

PIWI proteins are key regulators of transposon activity, especially in germline cells, where genomic integrity must be maintained to prevent mutations in future generations. PIWI proteins function by binding small RNA molecules called PIWI-interacting RNAs (piRNAs) [39,40,41,42], enabling the selective silencing of TE. Disruption of proper regulation of this process can lead to genetic mutations and compromised genome integrity. A crucial element in maintaining genomic stability is piRNA biogenesis, which ensures the silencing of TE in cells [41,42,43,44,45].

**Figure 2:**
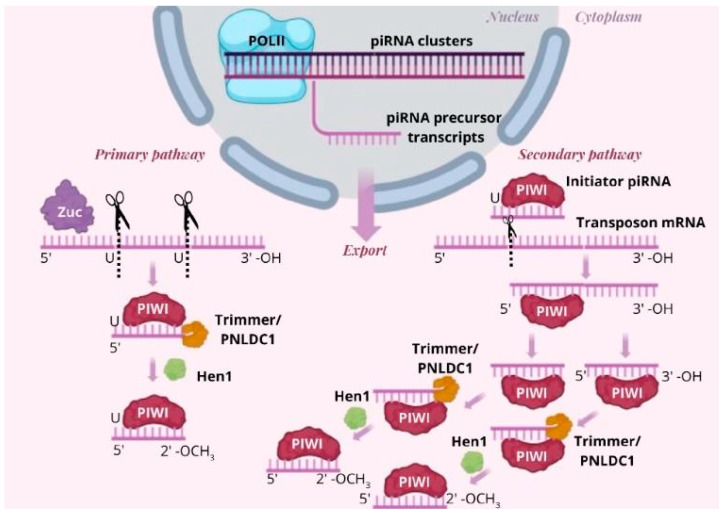
There are two piRNA biogenesis pathways: the primary pathway and the secondary pathway [25,46]. The primary pathway starts in the nucleus, where piRNA clusters are transcribed by RNA polymerase II (POL II). The resulting piRNA precursor is exported to the cytoplasm, where it connects with the Zucchini protein (Zuc), whose function is to split the transcribed strand at specific sites starting with a uracil (U) [6]. The 5′ end of the cut-out fragments connects with the PIWI protein (PIWI), creating a pre-piRNA in which the first nucleotide is uracil. The 3′ end undergoes methylation (by the methyltransferase Hen1) and is converted to 2′-OCH3 [36,37,38]. This mature piRNA is trimmed by the exonuclease Trimmer/PNLDC1 to produce mature initiator piRNAs, which is crucial in the secondary pathway. The initiator piRNA, the product of the primary pathway, begins the ping-pong cycle by slicing transposon mRNA into smaller sections, generating pre-pre-piRNAs. Each piece is bound by a PIWI protein creating pre-piRNA. The 3′ end of the generated molecule is trimmed by the exonuclease Trimmer/PNLDC1 and methylated by the methyltransferase Hen1 to finally produce the mature piRNA [25,46,47,48].

piRNA biogenesis is divided into two parts: the primary pathway and the secondary pathway [25,46]. The primary pathway begins in the nucleus, where the transcription of piRNA clusters takes place. The process then continues in the cytoplasm, where mature piRNA is formed. This mature piRNA later participates in the secondary pathway, which also occurs in the cytoplasm [25,46]. The primary pathway starts with the transcription of piRNA clusters (located in the genome) by RNA polymerase II (POL II) [26]. The resulting product, the piRNA precursor, is exported to the cytoplasm where it associates with the Zucchini protein (Zuc). Zuc functions as an endonuclease, cleaving the precursor strand at specific sites, typically starting with a uracil (U). The 5′ end of these cleaved fragments binds to PIWI proteins forming pre-piRNA, in which the first nucleotide is uracil. To generate mature piRNA, the 3′ end undergoes methylation by the methyltransferase Hen1 and is converted into 2′-OCH3 [36,37,38]. Next, the mature piRNA is trimmed by the Trimmer/PNLDC1 exonuclease to its final length, producing the mature initiator piRNA, which is essential for the secondary pathway [31]. The secondary pathway amplifies the piRNA pool and is strictly linked to the primary pathway. This process is known as the ping-pong cycle, named after its ability to regenerate piRNAs identical to the initiator molecules [25,44,46,47,48]. The initiator piRNA begins the ping-pong cycle by slicing the transposon mRNA into smaller fragments, generating the pre-pre-piRNAs. Each fragment is bound by a PIWI protein, forming pre-piRNA. The 3′ end of this molecule is further trimmed by the Trimmer/PNLDC1 exonuclease and methylated by the Hen1 methyltransferase, finally producing a mature piRNA. This mature piRNA is either identical or highly similar to the initiator piRNA, allowing it to initiate another round of the secondary pathway, creating a self-sustaining loop [25,31,36,37,38,44,46,47,48]. Additionally, some mature piRNAs are generated from the 3′ fragments of pre-pre-piRNAs that have undergone endonucleolytic cleavage. These piRNAs differ from the initiator piRNA, contributing to the diversity of the piRNA population. The key advantage of this system is that the products of one cycle can serve as initiators for the next, amplifying the piRNA biogenesis cycle and ensuring robust transposon silencing [25,46,47,48] (Figure 2).

## 4. Functions of piRNAs

### 4.1. Transposon Silencing

piRNA plays a crucial role in maintaining genome stability by repressing TEs at both the transcriptional and post-transcriptional levels, a process that depends on PIWI proteins [25,46,47]. At the transcriptional level, PIWI proteins bind to piRNA, while at the post-transcriptional level, they form the piRNA-induced silencing complex (piRISC) [25,46,47]. Post-transcriptional silencing is carried out by the cytoplasmic piRISCs, which use a similar mechanism to initiator piRNAs by slicing TEs. In contrast, nuclear piRISCs [25] take part in the transcriptional silencing by targeting transposons. This silencing pathway involves both binding mRNA at the site of transcription and disrupting genomic integrity via DNA recombination. Additionally, heterochromatinization not only prevents the expression of transposons but also reduces the recombination level, thereby further stabilizing the genome [25,42].

### 4.2. Maintaining Genome Stability

piRNAs control TEs through inducing the piRISC complex [48], thereby preventing the harmful activity of transposons within the genome. Uncontrolled activity of the transposons can lead to genome instability, giving rise to mutations, double-strand breaks, sterility, and even cancer development [25,49,50,51,52]. Recent research suggests that piRNA function differs between healthy and cancerous cells, raising an important question: Are cancers caused by piRNA dysregulation, or is piRNA dysregulation a consequence of cancer?

### 4.3. Epigenetic Regulation of Germline Cell Development

Epigenetic changes include modifications modulating gene expression without altering the genome’s nucleotide sequence. Among these DNA methylations, histone alterations [49,53] and non-coding RNAs, including PIWI-interacting RNAs (piRNAs), are particularly significant. DNA methylation is essential for the protection of the genome during gametogenesis and early embryonic development [49,54,55]. Notably, piRNAs facilitate the deposition of DNA methylation marks across large genomic regions in germ cells, implicating them as key guides in establishing these epigenetic signatures. Conversely, genome-wide DNA demethylation, which occurs after fertilization, is essential for restoring the totipotency of the zygote, erasing parental epigenetic memory, and preventing premature gamete specification. The deacetylation of histones—a key epigenetic process—is an important prerequisite for the chromosomal segregation during metaphase II [49,55]. Additionally, piRNAs modulate histone modifications, thereby playing a vital role in the intricate regulation of chromatin structure. Chromatin condensation, a result of histone modifications, provides a structural buffer for the dynamic changes occurring during gamete maturation [49]. Through sequence-specific base-pairing, piRNAs guide PIWI proteins to complementary targets, enabling transcriptional and post-transcriptional silencing of TEs [25,50]. A deficiency in piRNAs, PIWI proteins, or epigenetic regulation may lead to transposon reactivation and genomic disruption [25,50]. piRNAs regulate piRNA clusters at the transcriptional level by specific epigenetic modifiers, particularly those involved in DNA methylation and proteome regulation. Methylation of TEs involves de novo cytosine (C5) methylation, independent of prior methylation status. Moreover, piRNAs prevent inappropriate chromatin remodeling, thereby preserving genome integrity. Controlling transposons is critical across species due to their potential to induce harmful effects and genomic instability. In the germline, piRNAs mediate heritable transposon silencing primarily through de novo DNA methylation and heterochromatin formation. In contrast, somatic cells rely more heavily on siRNA-mediated pathways for transposon control. Thus, the piRNA pathway serves as a germline-specific and evolutionarily conserved mechanism for epigenomic protection [25,51]. Disruption of PIWI/piRNA signaling or failures in de novo DNA methylation are associated with transposon reactivation, genomic instability, and male sterility [25,50,51,52], highlighting their essential roles in germline integrity and the faithful transmission of genetic information.

The piRNA pathway orchestrates multiple layers of epigenetic regulation in the germline, including DNA methylation, histone modifications, and chromatin condensation. These processes are mediated through PIWI–piRNA complexes [25,50,51,52]. In fetal gonocytes, for example, nuclear PIWIL1–piRNA complexes are recruited to nascent transposon transcripts, where they facilitate the activity of epigenetic enzymes such as DNMT3A, DNMT3L, and SETDB1. These enzymes deposit CpG methylation and H3K9me3 marks at transposable element loci prior to meiosis, effectively locking these regions into a transcriptionally repressive chromatin state [25,50,51].

## 5. Role in Physiology and Pathophysiology

### 5.1. Regulatory Functions of piRNAs and PIWI Proteins in Germline

All functionally specialized cells are derived from stem cells, which are defined by their pluripotency and capacity for self-renewal. Primordial germ cells (PGCs) are formed during specific stages of early embryonic development. In the context of germline development, PGCs arise from embryonic stem cells (ESCs) and eventually differentiate into oocytes or sperm through the processes of oogenesis or spermatogenesis, respectively [55,56,57,58].

Oogenesis, the process of female germ cell maturation, begins during fetal development [54,55]. Oocytes are initially derived from PGCs, after which their development is arrested until puberty. Following a surge in luteinizing hormone (LH), oocyte maturation resumes through a series of transcriptional, physiological, and morphological changes. These include organelle maturation and epigenetic remodeling, such as de novo DNA methylation and histone modifications, which are crucial for successful meiotic progression and developmental competence [54,55,59,60].

Spermatogenesis, by contrast, is a continuous process occurring in the testes, aimed at generating large numbers of sperm necessary for male fertility [56,60,61]. Unlike oogenesis, male germline cells are produced throughout the reproductive lifespan in a cyclical sequence. This involves the differentiation of spermatogonia, which arise either from prospermatogonia during fetal development or from spermatogenic stem cells (SSCs) during adulthood [57,58,59,60,61]. Spermatogonia then give rise to type B spermatogonia, preleptotene spermatocytes, and ultimately spermatids. The elongation and terminal differentiation of early spermatids are essential for acquiring full functionality and fertilization capacity [60,61].

The PIWI-interacting RNA (piRNA) pathway is critical for protecting the germline genome from TE and plays an essential role in both male and female fertility. In females, three PIWI-subfamily proteins—PIWIL1, PIWIL2, and PIWIL3—are highly expressed during oogenesis and early embryogenesis. Deficiencies in PIWIL1 or PIWIL3 are associated with female infertility or reduced pregnancy rates. Although PIWIL2 is also abundantly expressed in primordial and primary-stage oocytes, its absence does not significantly impair fertility [59,62].

In males, distinct classes of piRNAs are produced at different developmental stages of germ cells, including “fetal piRNAs,” “postnatal piRNAs,” “pre-pachytene piRNAs,” “pachytene piRNAs,” and “hybrid piRNAs” [63]. These temporally regulated piRNA populations contribute to silencing TEs and orchestrating gene expression programs vital for spermatogenesis and germ cell integrity.

Disruption of the PIWI pathway halts spermatogenesis at various stages [50,64]. PIWIL4 deficiency reduces male germ cell numbers and impairs spermatogenesis due to defective transposon silencing [50,64]. Deletion of PIWIL2 results in the complete arrest of spermatogenesis, while PIWIL1 deletion causes spermatogenic failure at early stages [50,64]. Additionally, specific piRNA clusters are critical for proper sperm function; their deletion is associated with severe structural abnormalities such as sperm head dysmorphology and impaired motility, ultimately resulting in fertilization failure [50,64]. Oocytes fertilized with sperm lacking these piRNA clusters, via intracytoplasmic sperm injection (ICSI), develop into nonviable zygotes, underscoring the essential role of piRNAs not only in spermatogenesis but also in early embryonic development [64].

While embryonic stem cells (ESCs) are pivotal for generating all embryonic tissues, their defining features, self-renewal and pluripotency, are also shared by cancer stem cells (CSCs) [63,64]. Therefore, disruptions in ESC differentiation, such as altered epigenetic regulation, PIWI/piRNA pathway defects, transposon reactivation, or re-expression of key transcription factors such as OCT4, SOX2, or NANOG, can promote tumorigenesis [63,64,65,66]. Consequently, dysregulation of ESC differentiation mechanisms, including epigenetic alterations, defects in the PIWI/piRNA pathway, reactivation of transposable elements, or aberrant expression of transcription factors such as OCT4, SOX2, and NANOG, may contribute to tumorigenesis [63,64,65,66]. Despite growing evidence that PIWI proteins (PIWIL1–4) are frequently overexpressed in aggressive tumors, the specific roles of the piRNA pathway in somatic stem cells remain insufficiently characterized. Moreover, the complex regulatory crosstalk between piRNAs, microRNAs (miRNAs), and long non-coding RNAs (lncRNAs) represents a critical area for future research.

Emerging therapeutic strategies aim to restore transposon silencing in cancer cells through the delivery of synthetic piRNA mimics, or to target CSCs by inhibiting PIWI protein activity [50]. Although these approaches offer promising avenues for intervention, substantial challenges remain, particularly in achieving efficient and specific drug delivery, and in minimizing potential off-target effects that may compromise clinical applicability.

### 5.2. Aberrant Expression of piRNAs and PIWI Proteins as Biomarkers in Cancer

PIWI proteins are typically silenced in most healthy somatic tissues; however, their aberrant re-expression has been observed in various malignancies, including breast, colorectal, ovarian, and endometrial cancers [67,68,69]. This reactivation, particularly of PIWIL2, suggests that these tissues may exhibit unique regulatory landscapes permissive to PIWI gene expression [66,70]. Such dysregulation may result from alterations in epigenetic marks, including DNA methylation and histone modifications, which can either activate or silence PIWI genes in tumorigenic contexts [64,66,70]. Disruption of the piRNA pathway in cancerous or somatic cells leads to reactivation of TEs, posing a significant threat to genomic integrity. For instance, somatic retrotransposition of LINE-1 elements has been implicated in the inactivation of tumor suppressor genes, such as *APC* in colorectal cancer [71,72]. LINE-1 insertion into the factor VIII gene leads to hemophilia A [73,74]. Additionally, these insertions frequently induce DNA double-strand breaks, chromosomal rearrangements, and global epigenetic instability—hallmarks of many human cancers. Moreover, alterations in piRNA expression have been correlated with cancer progression and patient outcomes in several types of cancer [75,76,77,78,79,80]. In particular, piRNA-823 has attracted considerable attention in tumor biology. Overexpression of piRNA-823 has been associated with poor overall survival in patients with colorectal and renal cancers, as it promotes oncogenic processes by enhancing cell proliferation and suppressing apoptosis [75,76]. Conversely, downregulation of piRNA-823 results in impaired tumor cell growth, increased apoptotic activity, and reactivation of tumor suppressor genes previously silenced by aberrant methylation. This dual role highlights the potential of piRNA-823 not only as a prognostic biomarker for disease progression but also as a therapeutic target. Given its association with advanced tumor stages and its influence on fundamental cancer-related pathways, piRNA-823 could serve as a basis for personalized treatment strategies aimed at improving patient prognosis and quality of life [75,76].

Another piRNA of clinical interest is piRNA-651, which has been implicated in non-small-cell lung carcinoma (NSCLC), Hodgkin lymphoma, lung cancer, and breast cancer [77,78,79,80,81]. Elevated expression of piRNA-651 in NSCLC has been correlated with an increased mortality risk in NSCLC patients. Its overexpression in malignant tissues, compared to normal controls, promotes tumor cell proliferation, inhibits apoptotic pathways, and enhances metastatic potential [77,78,79]. Similarly, piRNAs have emerged as critical post-transcriptional regulators in gastric cancer, where expression patterns of specific piRNAs mirror those observed in colorectal and renal cancers, further underscoring the conserved roles of piRNAs in gastrointestinal malignancies [82,83].

Recent advances in high-throughput technologies, such as next-generation sequencing (NGS), have enabled the detection of piRNAs in biofluids, expanding their potential utility as non-invasive diagnostic biomarkers. For instance, in Wilms tumor, several circulating piRNAs—including piR-hsa-1,913,711, piR-hsa-28,190, piR-hsa-28,849, piR-hsa-28,848, and piR-hsa-28,318—were significantly downregulated in serum samples from patients compared to healthy controls, highlighting their potential for early cancer detection via liquid biopsy [84].

As the scientific community’s interest in small non-coding RNAs continues to grow, piRNAs have become a focal point for understanding cancer-associated gene regulatory networks, particularly those reflective of stem cell-like properties [50,85,86,87]. In this context, aberrant expression of PIWI proteins, especially PIWIL2, has been documented in a wide range of tumors, including colorectal carcinoma, gastric cancer, prostate cancer, breast cancer, gliomas, soft tissue sarcomas, cervical cancer, and hepatocellular carcinoma [50,88,89,90,91,92,93,94,95]. Although PIWIL2 overexpression is associated with distant metastasis in a minority of cases, it does not yet qualify as a definitive prognostic marker for metastatic progression. Nevertheless, reduced PIWIL2 expression correlates positively with increased overall survival across multiple cancer types, suggesting its potential as a general prognostic indicator [70].

The oncogenic or tumor-suppressive roles of piRNAs and PIWI proteins appear to be highly context-dependent. Functional studies confirm that PIWIL1 and PIWIL2 frequently act as oncogenic drivers in cancers such as glioma, lung, and colorectal carcinoma. Knockdown of these proteins inhibits tumor cell proliferation, induces apoptosis, and halts cell cycle progression. Likewise, specific piRNAs, including piR-823 and piR-651, have been shown to contribute to tumorigenesis by modulating key signaling pathways (Table 1). These findings collectively suggest that the piRNA-PIWI axis is not only a hallmark of germline genome defense but also a critical regulator of somatic oncogenic processes.

### 5.3. Epigenetic Regulation by piRNAs: A Non-Complementary Mechanism of Gene Expression Control

Epigenetic regulation is defined by the potentially reversible modulation of gene expression in response to environmental stimuli. This dynamic control mechanism holds significant therapeutic promise, particularly for mitigating the adverse effects of environmental exposures on human health. One emerging strategy involves targeted gene methylation via repurposing the germline’s transposon-silencing machinery—specifically the piRNA-PIWI axis—as a more precise alternative to broad-spectrum epigenetic modifiers [50,91,96].

piRNAs exert epigenetic influence through mechanisms distinct from those of mRNAs. Unlike mRNAs, piRNAs do not require perfect complementarity to their target sequences, allowing them to regulate gene expression through a unique mode of sequence recognition. This flexibility enables the selective coordination of gene expression at specific developmental or cellular time points. However, in cancer cells, frequent hypomethylation or aberrant hypermethylation disrupts piRNA expression, impairing the piRNA-mediated transposon silencing machinery and contributing to genomic instability [50,91].

In transformed or mutated cells, piRNAs participate in the regulation of essential oncogenic processes, including proliferation, apoptosis, metastasis, and invasion. Notably, the piRNA/PIWI signaling pathway has also been implicated in the development of chemoresistance. Its involvement in modulating tumor cell responses to therapy underscores its potential as a therapeutic target to overcome drug resistance [50,97]. The PIWI protein family plays a central role in these processes due to its involvement in RNA-mediated gene silencing, stem cell self-renewal, and translational control across multiple species. By associating with piRNAs, PIWI proteins form functional complexes that direct epigenetic remodeling through DNA methylation and histone modifications, contributing to both germline maintenance and oncogenic transformation [50,91].

In conclusion, although significant progress has been made in elucidating the functions of piRNAs and PIWI proteins in maintaining genome integrity within germline and cancer contexts, the precise molecular mechanisms linking these factors to carcinogenesis, chemotherapy resistance, fertility, and broader epigenetic regulation remain incompletely understood. Continued investigation into these pathways is essential for advancing therapeutic strategies targeting the piRNA-PIWI axis in both reproductive health and oncology.

**Table 1:** Summary of PIWI proteins’ role in physiology and pathophysiology.

PIWI Protein	Role in Oogenesis [59,66]	Role in Spermatogenesis [61,63,64]	Role in Carcinogenesis [50,83,85,92,93]
PIWIL1	Highly expressed. Involved in regulating transposon silencing and genomic stability. Crucial for proper oocyte development at the blastomere level.	Determines the proper sperm phenotype, ensuring proper spermatogenic development at the early stages.	Not described.
PIWIL2	No apparent influence on female fertility.	Presence determines the occurrence of the spermatogenesis process.	Determines occurrence of the distant metastasis.
PIWIL3	Highly expressed. Involved in regulating litter size and pregnancy rate.	Not found in spermatogenic cells.	Not described.
PIWIL4	Not found in oocytes cells.	Involved in regulating male germ cell numbers and spermatogenesis arrest.	Crucial for the proliferation and apoptosis process of several cancers.

## 6. Summary

PIWI-interacting RNAs (piRNAs) and PIWI proteins are critical regulators of epigenetic processes, originally discovered through studies focused on transposon silencing in the germline. Although research into the piRNA/PIWI axis remains ongoing, current findings have significantly advanced our understanding of genome stability, epigenetic control, and germ cell development. Notably, dysregulation of this pathway has been implicated in germline defects, infertility in both sexes, and a variety of epigenetic disorders.

Beyond the reproductive system, emerging evidence indicates that the aberrant activity of the PIWI/piRNA pathway contributes to cancer development and progression. Disruptions in this regulatory axis may lead to uncontrolled cell proliferation, compromised DNA repair, and chromatin remodeling—hallmarks of oncogenesis. However, it remains unclear whether these molecular changes represent primary drivers or secondary consequences of malignant transformation. Resolving this question is critical for advancing the field.

Future research holds considerable promise for clarifying the precise mechanistic roles of piRNAs and PIWI proteins in cancer biology. A more comprehensive understanding could facilitate the development of novel, targeted treatment strategies with significant therapeutic potential. Two priority areas merit immediate focus: The first is the development of piRNA-based biomarkers. Due to their high stability in biological fluids, piRNAs are excellent candidates for non-invasive diagnostic tools. Circulating piRNA profiles, detectable in liquid biopsies such as blood or urine, may serve as sensitive biomarkers for early cancer detection, prognosis, and disease monitoring. This diagnostic utility may also extend to reproductive medicine, where piRNAs could aid in the assessment of male infertility. Second is the therapeutic targeting of the PIWI/piRNA pathway. The use of RNA interference or other RNA-based therapies to modulate PIWI protein expression offers a novel approach for disrupting tumor-promoting pathways. These interventions may effectively inhibit tumor growth or sensitize cancer cells to existing treatments.

In summary, advancing our understanding of piRNAs and PIWI proteins may not only illuminate fundamental aspects of genome regulation but also unlock innovative diagnostic and therapeutic applications in oncology and reproductive health. Such progress is vital for improving patient outcomes and developing precision medicine approaches tailored to individual molecular profiles [38,39].

## Data Availability

No new data were created or analyzed in this study.
